# Retinol Saturase Knock-Out Mice are Characterized by Impaired Clearance of Apoptotic Cells and Develop Mild Autoimmunity

**DOI:** 10.3390/biom9110737

**Published:** 2019-11-13

**Authors:** Zsolt Sarang, Tibor Sághy, Zsófia Budai, László Ujlaky-Nagy, Judit Bedekovics, Lívia Beke, Gábor Méhes, Gábor Nagy, Ralph Rühl, Alexander R. Moise, Krzysztof Palczewski, Zsuzsa Szondy

**Affiliations:** 1 Department of Biochemistry and Molecular Biology, Faculty of Medicine, University of Debrecen, Debrecen H-4012, Hungary; sarang@med.unideb.hu (Z.S.); budai.zsofia@med.unideb.hu (Z.B.); 2 Department of Dental Biochemistry, Faculty of Dentistry, University of Debrecen, Debrecen H-4012, Hungary; saghy.tibor@med.unideb.hu; 3 Department of Biophysics and Cell Biology, Faculty of Medicine, University of Debrecen, Debrecen H-4012, Hungary; lnagy@med.unideb.hu; 4 Department of Pathology, Faculty of Medicine, University of Debrecen, Debrecen H-4012, Hungary; bedekovics.judit@med.unideb.hu (J.B.); beke.livia@med.unideb.hu (L.B.); gabor.mehes@dote.hu (G.M.); 5 Department of Laboratory Medicine, Faculty of Medicine, University of Debrecen, Debrecen H-4012, Hungary; nagy.gabor@unideb.hu; 6 Paprika Bioanalytics BT, Debrecen H-4002, Hungary; ralphruehl@web.de; 7 Medical Sciences Division, Northern Ontario School of Medicine, Sudbury, ON P3E 2C6, Canada; amoise@nosm.ca; 8 Gavin Herbert Eye Institute and the Department of Ophthalmology, University of California, Irvine, CA 92697, USA; kpalczew@uci.edu

**Keywords:** retinol saturase, efferocytosis, MFG-E8, macrophage, autoimmunity, neuropeptide Y

## Abstract

Apoptosis and the proper clearance of apoptotic cells play a central role in maintaining tissue homeostasis. Previous work in our laboratory has shown that when a high number of cells enters apoptosis in a tissue, the macrophages that engulf them produce retinoids to enhance their own phagocytic capacity by upregulating several phagocytic genes. Our data indicated that these retinoids might be dihydroretinoids, which are products of the retinol saturase (RetSat) pathway. In the present study, the efferocytosis of RetSat-null mice was investigated. We show that among the retinoid-sensitive phagocytic genes, only transglutaminase 2 responded in macrophages and in differentiating monocytes to dihydroretinol. Administration of dihydroretinol did not affect the expression of the tested genes differently between differentiating wild type and RetSat-null monocytes, despite the fact that the expression of RetSat was induced. However, in the absence of RetSat, the expression of numerous differentiation-related genes was altered. Among these, impaired production of MFG-E8, a protein that bridges apoptotic cells to the α_v_β_3_/β_5_ integrin receptors of macrophages, resulted in impaired efferocytosis, very likely causing the development of mild autoimmunity in aged female mice. Our data indicate that RetSat affects monocyte/macrophage differentiation independently of its capability to produce dihydroretinol at this stage.

## 1. Introduction

Timed induction of apoptosis and the proper clearance of apoptotic cells play a crucial role in the maintenance of tissue homeostasis. The human body collectively turns over about 200–300 billion cells every day. Dying cells are normally taken up by macrophages by simultaneously using several phagocytic receptors. These receptors recognize cell surface changes on the apoptotic cells through either direct apoptotic cell–phagocyte interactions or serum opsonizing proteins that bridge apoptotic ligands and the phagocyte receptors [1]. The appearance of phosphatidylserine (PS) on the surface of the apoptotic cell is the most ubiquitous indicator of apoptosis [2]. It is recognized by BAI1 [3], stabilin-2 [4], and Tim-4 [5] directly, while other phagocytic receptors utilize the bridging molecule milk fat globule EGF-factor 8 (MFG-E8) [6], thrombospondin-1 [7], Gas6, Protein S [8], or complement 1q (C1q) [9] for PS binding.

Macrophages can both express phagocytic receptors and release bridging molecules for recognition and engulfment of apoptotic cells. Some of these receptors, such as Tim-4 [10] or CD14 [11], mediate tethering, while other receptors, such as CD36 [12], Mer tyrosine kinase (Mertk) [13], stabilin-2 [4], BAI1 [3], or integrin β_3_ with is coreceptor transglutaminase 2 (TG2) [14], trigger two evolutionally conserved parallel signaling pathways that initiate cytoskeletal reorganization via activating the low molecular weight GTPase Rac1 [15]. Different macrophage populations were shown to use different phagocytic receptors or bridging molecules for efferocytosis; thus, peritoneal macrophages express high levels of Tim-4, while tingible body macrophages secrete MFG-E8 [5,16].

Macrophages are exposed to varying numbers of apoptotic cells in vivo, and therefore they need a mechanism to prepare them, when required, for increased levels of apoptotic cell uptake. The receptors, which sense the amount of apoptotic cell material engulfed and enhance the phagocytic capacity of macrophages in response by inducing the expression of various efferocytosis-related molecules, are the lipid-sensing receptors, such as the liver X receptor (LXR) [17] and the peroxisome proliferator-activated receptors (PPAR)s [18,19]. All these receptors function as retinoid X receptor (RXR) heterodimers.

Previous work in our laboratory has shown that activation of these lipid-sensing receptors enhances retinoic acid synthesis in macrophages [20], which partially mediates their effect on the efficacy of apoptotic cell phagocytosis [21]. However, in the apoptosing thymus, we were unable to demonstrate production of the classically known retinoic acids, despite the increased retinoic acid synthesis that we detected with the help of the RARE LacZ mouse, in which the expression of the LacZ reporter gene is fully dependent on endogenous retinoic acid synthesis [20]. Instead, we found a compound produced in a retinaldehyde dehydrogenase-dependent manner, which, based on its molecular weight, could have been a dihydroretinol derivative. Simultaneously, we detected induction of the retinol saturase enzyme (RetSat) [21].

Retinol saturase performs a stereospecific saturation of the C13–C14 double bond of all-*trans*-retinol to generate (13*R*)-all-*trans*-13,14-dihydroretinol. This compound is found in cells expressing retinol saturase and in the livers of mice fed with retinyl palmitate [22]. All-*trans*-13,14-dihydroretinol (dihydroretinol) is oxidized in vivo to all-*trans*-13,14-dihydroretinoic acid, a highly selective agonist of the retinoic acid receptor (RAR), and to 9-*cis*-13,14-dihydroretinoic acid, a highly selective agonist of the RXR receptor [23]. In fact, it is suggested that 9-*cis*-13,14-dihydroretinoic acid might be the long-sought physiological RXR ligand [24]. However, it was also demonstrated that RetSat might perform other biological functions as well, as not all the consequences of its loss could be restored by dihydroretinol administration [25,26,27]. In the present paper, we describe a new efferocytosis-related phenotype of RetSat knock-out mice.

## 2. Materials and Methods

### 2.1. Reagents

All reagents were obtained from Sigma-Aldrich (Budapest, Hungary) except when indicated otherwise.

### 2.2. Experimental Animals

The experiments were carried out with 4 week, 2–4 month or 1 year old C57BL/6J RetSat^+/+^ mice and their RetSat^−/−^ littermates [28]. For determining gene expression in the thymus or thymic cell composition, 4 week old mice were injected intraperitoneally with either 0.3 mg dexamethasone acetate (DEX) dissolved in dimethyl sulfoxide (DMSO) or vehicle alone, or exposed to 5 Gy irradiation. For determining the long-term effect of DEX treatment in the thymus, 4 week old mice were injected with 1 mg/kg DEX daily for 6 days, then exposed to 0.5 Gy irradiation as described by Lauber et al. [29]. Mice were maintained in specific pathogen-free conditions in the Central Animal Facility, and all animal experiments were approved by the Animal Care and Use Committee of the University of Debrecen (DEMÁB).

### 2.3. Flow Cytometry Analysis of Freshly Isolated Thymocytes

At the indicated time points after the various treatments of mice, thymocytes were isolated, washed twice, and resuspended in ice-cold PBS before staining either with phycoerythrin (PE)-labeled anti-CD4 and Cy5-conjugated anti-CD8 (Pharmingen, San Diego, CA, USA) antibodies or with fluorescein isothiocyanate (FITC)-labeled annexin V in binding buffer. Cell-bound fluorescence was analyzed in a blinded fashion using a FACSCalibur (Beckton Dickinson).

### 2.4. Retinoid Measurement by High-performance Liquid Chromatography Mass Spectrometry (HPLC/MS/MS)

Four-week-old RetSat-null mice were injected i.p. with either 0.3 mg dexamethasone acetate (Dex) dissolved in DMSO alone or with *N*,*N*-diethylaminobenzaldehyde (DEAB) (0.24 mg/g body weight) or vehicle. After 24 h, thymi were removed in the dark, snap-frozen in liquid nitrogen, and stored at −70 °C. Concentrations of retinoic acids were determined in mouse thymi by our HPLC-MS-MS method [30]. In summary, 100 mg of the thymic samples (if samples were under 100 mg, water was added to attain a 100 mg sample) were diluted with a threefold volume of isopropanol, the tissues were minced using scissors, vortexed for 10 s, put in an ultrasonic bath for 5 min, shaken for 6 min, and centrifuged at 13,000 rpm in a Heraeus BIOFUGE Fresco (Kendro Laboratory Products, East Coast, Southern, US) at +4 °C. After centrifugation, the supernatants were dried in an Eppendorf concentrator 5301 (Eppendorf, Germany) at 30 °C. The dried extracts were resuspended with 60 μL of methanol, vortexed, shaken, diluted with 40 μL of 60 mM aqueous ammonium acetate solution, transferred into the autosampler, and subsequently analyzed using HPLC-MS-MS equipment. In addition, we focused on detecting novel dihydro-retinoic acid derivatives, and we switched our MS-MS to single ion recording (SIR) mode, focusing on 303 m/z signals in relative intensity.

### 2.5. Thymocyte Apoptosis in Vitro 

Isolated thymocytes (10^5^ cells/mL) were cultured in RPMI 1640 medium supplemented with 5% fetal bovine serum (FBS), 2 mM glutamine, 1 mM Na-pyruvate, and 5 × 10^−5^ M 2-mercaptoethanol at 37 °C/5% CO_2_. Apoptosis was induced by addition of 0.1 μM DEX. After 6 h, the extent of cell death was determined by Annexin V-FITC labeling of apoptotic cells. Cell-bound fluorescence was analyzed by FACSCalibur (Beckton Dickinson).

### 2.6. Bone-Marrow-Derived (BMDM), Peritoneal, or Thioglycolate-elicited Macrophage Generation, Cell Culture, and Treatment

Bone marrow progenitors were obtained from the femur of 2 to 4 month old mice by lavage with sterile physiological saline. Cells were differentiated for 5 days in DMEM medium supplemented with 10% conditioned medium derived from L929 cells, as a source for macrophage colony stimulating factor (M-CSF), 2 mM glutamine, 100 U/mL penicillin, and 100 mg/mL streptomycin at 37 °C in 5% CO_2_. Non-adherent cells were washed away every second day. BMDMs from both wild type and RetSat-null mice were collected at the end of differentiation to determine their total mRNA expression levels and every day during their differentiation alone or in the presence of all-*trans*-13,14-dihydroretinol (Santa Cruz Biotechnology, Heidelberg, Germany) for qRT-PCR analysis of the expression of their various genes. Tissue-resident peritoneal macrophages were isolated by peritoneal lavage with RPMI 1640 medium. For generating thioglycolate-elicited peritoneal macrophages, mice were treated with 4 mL of 4% thioglycolate (in PBS), and four days later, macrophages were collected by peritoneal lavage. Peritoneal macrophages were then seeded on 24-well plates in RPMI 1640 medium containing 10% fetal calf serum (FCS) (1 × 10^6^ cells). BMDMs or tissue-resident peritoneal macrophages were treated with various compounds as indicated in the figure legends, and their phagocytic capacity or mRNA expressions were also determined.

### 2.7. In Vitro Apoptotic Cell Phagocytosis

BMDMs or peritoneal macrophages were stained for 24 h with 10 μM 5(6)-carboxyfluorescein diacetate (CFDA-SE) (Invitrogen, Carlsbad, CA, USA). To generate apoptotic thymocytes, the thymus was collected from 4 week old C57BL/6 mice, thymocytes were isolated and cultured for 24 h (10^7^ cells/mL) in RPMI 1640 medium supplemented with 2 mM glutamine, 100 U/mL penicillin, 100 mg/mL streptomycin, and 2.5 μM Deep Red dye (Invitrogen, Carlsbad, CA, USA) in the absence of serum. The percentage of apoptotic thymocytes determined by annexin V labeling was ≥ 80%. Stained apoptotic thymocytes were added to the macrophages in a 5:1 (apoptotic cells:macrophage) ratio for 1 h. After coculture, apoptotic cells were washed away and macrophages were detached by trypsinization. For long-term phagocytosis assays, macrophages were first exposed to unstained apoptotic cells for 5 h and then to the stained apoptotic cells for an additional hour. Cells were analyzed using either FACSCalibur or confocal microscopy.

### 2.8. Confocal Microscopy

Peritoneal and bone-marrow-derived macrophages from wild type (WT) and RetSat^−/−^ mice were plated in 8-well chamber slides (3 × 105/well) (Gräfelfing, Germany). Phagocytosis assays were carried out as described above. Macrophages were then washed and fixed in 1% paraformaldehyde. Calculations and statistical analyses were based on microscopic images. Fluorescence confocal images were taken with a Zeiss LSM 880 invert microscope using appropriate excitations and emission filters corresponding to the different fluorescence markers (objective: C-Apochromat 40x/1.2 W Korr; 488 nm excitation with 493–600 nm emission range; 633 nm excitation with 638–755 nm emission range). Pixel size and sampling was fit to an appropriate wavelength and followed the Nyquist rate. A high resolution tile scan automatic focus readjusting system was used. Further image analysis was undertaken in Zeiss Zen and ImageJ to determine the number of ingested apoptotic cells.

### 2.9. mRNA Sequencing

To obtain global transcriptome data of wild type and RetSat-null BMDMs, high-throughput mRNA sequencing analysis was performed on an Illumina sequencing platform. The quality of total RNA samples was checked on an Agilent BioAnalyzer using the Eukaryotic Total RNA Nano Kit according to the manufacturer’s protocol (Agilent, Santa Clara, CA, USA). Samples with RNA integrity number (RIN) values > 7 were accepted for the library preparation process. RNA-Seq libraries were prepared from total RNA using the TruSeq RNA Sample preparation kit (Illumina) according to the manufacturer’s protocol. Briefly, poly-A RNAs were captured by oligo-dT conjugated magnetic beads, and then the eluted mRNAs were fragmented at 94 ℃. First-strand cDNA was generated by random priming reverse transcription, and after a second-strand synthesis step, double-stranded cDNA was generated. After repairing ends, A-tailing and adapter ligation steps, adapter ligated fragments were amplified in enrichment PCR, and finally, sequencing libraries were generated. Sequencing runs were executed on an Illumina HiSeq2500 instrument using single-end 50 bp sequencing. Gene ontology (GO) term enrichment analysis of the 117 differentially expressed genes was carried out using Search Tool for the Retrieval of Interacting Genes v10 (STRING), covering both physical interactions and functional associations between proteins (JensenLab, https://string-db.org/).

### 2.10. Quantitative Real-Time Polymerase Chain Reaction (qRT-PCR) Analysis of mRNA Expression

Total RNA was isolated from thymi, BMDMs, or peritoneal cells of wild type or RetSat^−/−^ mice using the TRI reagent according to the manufacturer’s guidelines (ThermoFisher, Waltham, MA, USA). Total RNA was reverse transcribed into cDNA using a High Capacity cDNA Reverse Transcription Kit (Life Technologies, Budapest, Hungary) according to the manufacturer’s instruction. qRT-PCR was carried out in triplicate using pre-designed FAM-labeled MGB assays (Life Technologies, Budapest, Hungary) on a Roche LightCycler LC 480 real-time PCR instrument. Relative mRNA levels were calculated using the comparative C_T_ method and were normalized to β-actin mRNA.

### 2.11. Anti-Nuclear Antibody Detection by Indirect Immunofluorescence Assay

Anti-nuclear antibodies were detected on HEp-2 cells using an indirect immunofluorescence assay kit (Euroimmun GmbH, Lübeck, Germany) according to the manufacturer’s instructions, with the only modification of replacing the original conjugate solution with a goat anti-mouse IgG (H+L) antibody labelled with Alexa Fluor 488 (A-11001, Thermo Fisher Scientific, Rockford, USA, 1:400 dilution). The fluorescence pattern and intensity of each well were read visually on a fluorescence microscope with an LED light source (Eurostar II Plus). The fluorescence intensity was recorded on a semi-quantitative scale (negative, 1+, 2+, 3+, 4+ positive).

### 2.12. Anti-dsDNA Antibody ELISA

Samples were tested for anti-dsDNA antibodies using the immunometric, direct ELISA test kit (ORG 604) from Orgentec Diagnostika GmbH, Mainz, Germany. In order to detect mouse autoantibodies, the original conjugate was substituted with a sheep anti-mouse IgG antibody conjugated with peroxidase (1:10000 dilution). After stopping the chemical reaction in the wells, the optical densities (OD) were read at 450 nm on an ETI-MAX 3000 ELISA processor.

### 2.13. Caspase-3 Immunohistochemistry

Spleens were removed from 1 year old wild type or RetSat^−/−^ mice and fixed in formaldehyde (4% in phosphate buffer) for 1 day. Dehydration was carried out before embedding into paraffin according to standard protocol. After paraffin became solid, blocks were cut with a microtome to obtain 3–4 micrometer thick sections. After the deparaffination and hematoxylin and eosin (HE) staining, immunohistochemical staining was performed using anti-Cleaved Caspase-3 (Cell Signaling Technology, Inc, Global Headquarters USA) primary monoclonal antibody at a dilution of 1:300 and an horse radish peroxidase (HRP)-labelled polymer anti-rabbit secondary antibody (Dako, Glostrup, Denmark). The intensity and distribution of immunostaining was assessed by light microscopy (Leica DM2500 microscope, DFC 420 camera and Leica Application Suite V3 software; Leica).

### 2.14. Detection of IgM-Containing Immune Complexes

The kidney was removed from 1 year old RetSat^+/+^or RetSat^−/−^ mice and horizontally sliced, and then tissue blocks were snap-frozen with isopentane in liquid nitrogen. Four-micrometer cryosections were cut, fixed in cold acetone, and incubated with FITC-labeled anti-mouse IgM diluted in PBS (1:40). After rinsing in PBS, slides were mounted in buffered glycerol. Immunostaining was assessed by light microscopy (Leica DM2500 microscope, DFC 420 camera and Leica Application Suite V3 software; Leica).

### 2.15. Determination of Serum Urea Concentration

The method described by Rahmatullah and Boyde was used [31].

### 2.16. Statistical Analysis 

Data are presented as mean ± SD for all data. All statistical analyses were performed using GraphPad Prism 6.01 and a *p*-value < 0.05 was considered as significant and is indicated by asterisk (*). For differences between 2 groups, the 2-tailed unpaired Student’s t-test was used, and for comparisons of n > 2 groups, the one-way ANOVA (with Turkey’s multiple comparisons) test was used.

## 3. Results

### 3.1. Loss of Retinol Saturase Does Not Affect the Induction of Retinoid-Regulated Genes or the Thymic Apopto-Phagocytosis Program

**Figure 1:**
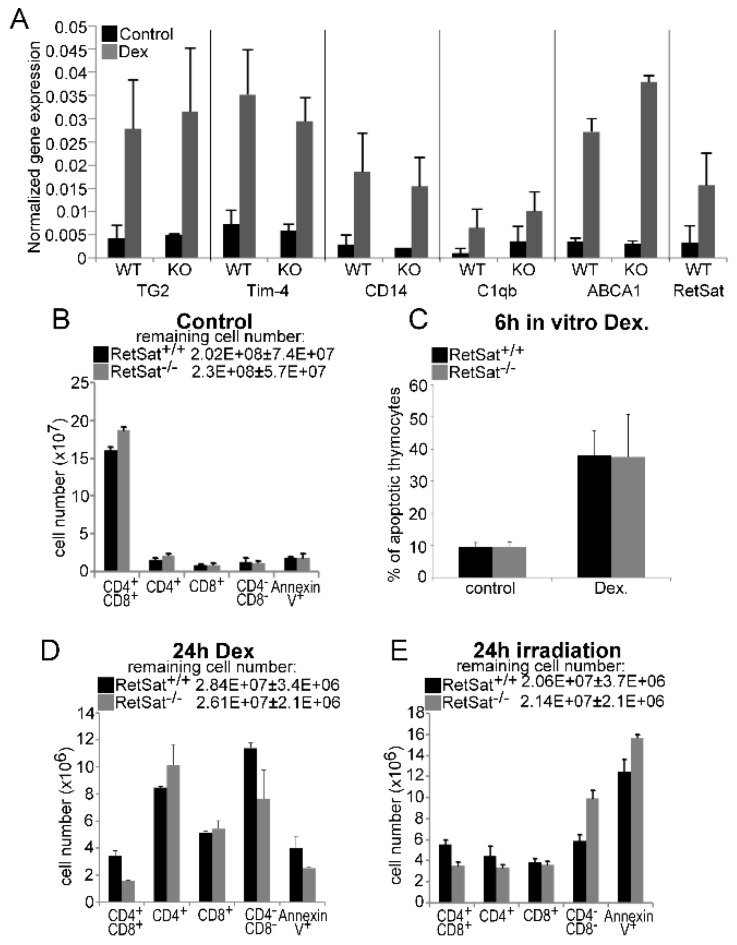
Loss of retinol saturase (RetSat) does not affect the expression of retinoid-regulated efferocytosis-related genes or the thymic apopto-phagocytosis program in mice. (**A**) 4 week old mice were injected intraperitoneally with 0.3 mg dexamethasone acetate (DEX) or vehicle to induce thymocyte apoptosis. After 24 h, thymi were collected and gene expression was determined by qRT-PCR (n = 4) using β-actin as a normalizing gene. WT, wild type; KO, knock-out. Results are expressed as mean ± SD (n = 4). (**B**) The number of various thymocyte cell populations in the thymi of 4 week old wild type and RetSat-null mice. Results are expressed as mean ± SD (n = 4). (**C**) Percentage of annexin-V-positive thymocytes 6 h after apoptosis induction by 1 μM DEX in vitro. (**D**,**E**) The number of various thymocyte cell populations in the thymi of 4 week old wild type and RetSat-null mice at 24 h after exposure to 0.3 mg DEX (**D**) or 5 Gy irradiation (**E**). Results are expressed as mean ± SD (n = 4).

Our previous studies indicated that in macrophages, during engulfment of apoptotic cells the expression of at least 5 efferocytosis-related genes (TG2, CD14, C1qb, Tim-4, and ABCA1) is induced in a retinoid-dependent manner; the expression of these genes is enhanced in the apoptosing thymus, but the classical retinoic acids could not be detected, unlike a potential dihydroretinol derivative [21]. Therefore, we decided to determine whether the induction of the same genes is affected in the absence of RetSat in the apoptosing thymus. As seen in Figure 1A, induction of none of the tested genes was altered by the loss of RetSat in the apoptosing thymus. In addition, we could detect the appearance of the same retinaldehyde dehydrogenase-dependent retinoid peak of unknown identity in the apoptosing thymus that we suspected to be a dihydroretinol derivative (data not shown). Thus, we concluded that either RetSat-related retinoids do not play a determining role in the induction of retinoid-related genes, or in the absence of RetSat, they can be replaced by other retinoids in the mouse thymus. 

Since the apopto-phagocytosis program plays a crucial role in shaping the T cell repertoire [32,33] and retinoids were shown by us to influence the thymic T cell selection processes [34], we investigated whether loss of RetSat affects the composition of the thymus. However, as shown in Figure 1B, loss of RetSat did not affect the cellularity or the size of the various thymocyte populations. Since RetSat was induced in engulfing macrophages, we also tested whether loss of RetSat affected the ability of macrophages to clear apoptotic thymocytes in vivo. RetSat-null thymocytes induced to die in vitro entered apoptosis with the same speed (Figure 1C). Similarly, induction of cell death either by dexamethasone or by γ-irradiation did not significantly affect thymocyte loss or the percentage of annexin-V-positive uncleared thymocyte population (Figure 1D,E), indicating that neither the apoptosis of thymocytes nor their phagocytosis was significantly affected in vivo by the loss of RetSat.

### 3.2. RetSat-Null Macrophages Are Characterized by Impaired Long-Term Phagocytosis

**Figure 2:**
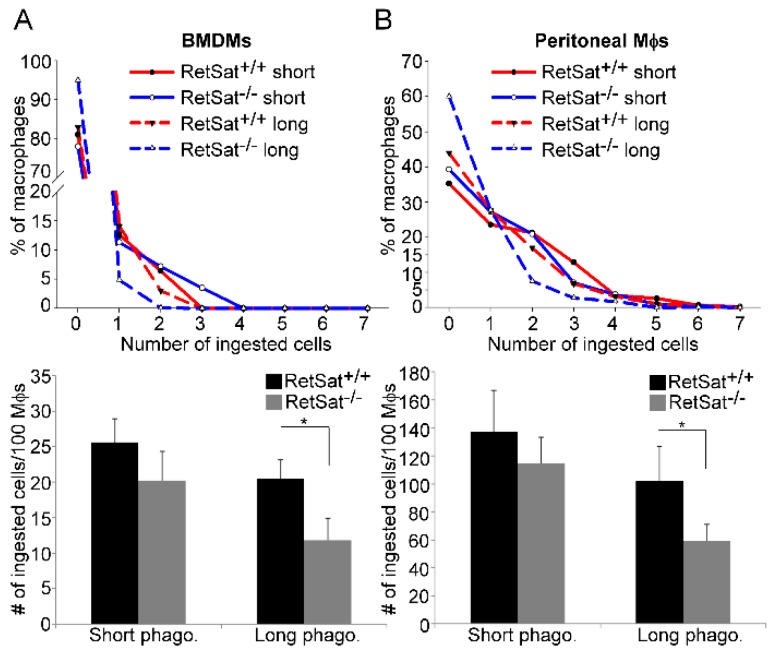
Loss of RetSat delays the long-term efferocytosis of both bone-marrow-derived macrophages (BMDMs) and peritoneal macrophages. 5(6)-carboxyfluorescein diacetate (CFDA-SE)-labelled (**A**) BMDMs or (**B**) peritoneal macrophages were exposed to Deep-Red-dye-labelled apoptotic thymocytes for 1 h (in a 1:5 macrophage—target-cell ratio) either immediately (short-term phagocytosis) or after 5 h of engulfing non-labelled apoptotic thymocytes. The number of ingested cells was determined by confocal microscopy. Upper panels show one representative experiment. Total number of ingested cell results in lower panels are expressed as mean ± SD (n = 3), * *p* < 0.05.

Since in vivo various macrophage populations use different phagocytic receptors and bridging molecules for efferocytosis [5,16], we decided to test the in vitro efferocytotic capacity of RetSat-null peritoneal and bone-marrow-derived macrophages (BMDMs). As shown in Figure 2A,B, short term phagocytosis determined following a 1 h uptake of apoptotic cells was not affected by the loss of RetSat. However, if phagocytosis was determined after 5 h of continuous efferocytosis, the phagocytic capacity of both types of RetSat-null macrophages was decreased as compared to their wild type control. These data indicate that RetSat contributes to the efficient efferocytosis of apoptotic cells.

### 3.3. RetSat-Null Macrophages Express Less MFG-E8

**Figure 3:**
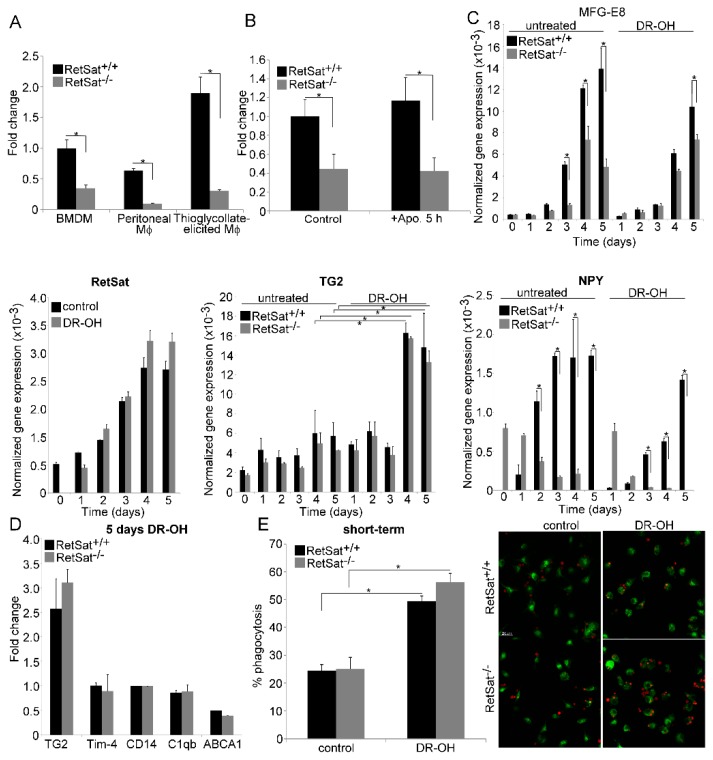
Loss of RetSat leads to decreased MFG-E8 mRNA expression in various macrophages. (**A**) Expression of MFG-E8 in wild type and RetSat-null BMDMs, peritoneal macrophages, and thioglycolate-elicited macrophages as compared to wild type BMDMs. These macrophages were generated as described in Materials and Methods. (**B**) Relative MFG-E8 mRNA expression of wild type and RetSat-null BMDMs before and after 5 h efferocytosis as compared to the non-engulfing wild type BMDMs. (**C**) Normalized mRNA expressions of RetSat, MFG-E8, TG2, and NPY during differentiation of wild type and RetSat-null monocytes in the presence and absence of 1 μM all-*trans*-13,14- dihydroretinol (DR-OH). (**D**) Induction of retinoid-sensitive efferocytosis genes at the end of the 5 day monocyte differentiation in wild type and RetSat-null cells in the presence of 1 μM all-*trans*-13,14- dihydroretinol, as compared to the vehicle-treated control cells. mRNA expressions in all these experiments were determined by qRT-PCR using β-actin as a normalizing gene. Results are expressed as mean ± SD (n = 4), * *p* < 0.05. (**E**) Short-term phagocytosis of wild type and RetSat-null BMDMs differentiated in the presence or absence of 1 μM all-*trans*-13,14-dihydroretinol. Percentage of engulfing macrophages was determined by flow cytometry analysis. Results are expressed as mean ± SD (n = 4), * *p* < 0.05. One representative confocal image is also shown. CFDA-SE-labelled macrophages appear as green cells, while Deep Red dye-labelled apoptotic thymocytes appear as red cells. Engulfed apoptotic thymocytes appear with orange color, as the green and red colors overlap.

To determine the reason behind the long-term phagocytic defect, we applied total RNA sequencing and identified 117 differentially expressed genes (DEGs) between RetSat^+/+^ and RetSat^−/−^ BMDMs (based on at least a 1.5-fold change and corrected *p* value < 0.05). A total of 59 transcripts showed decreased gene expression and 58 transcripts showed increased gene expression in the RetSat^−/−^ cells. The list of DEGs is shown in Table 1. The mean fold change (FC) of decreased and increased transcripts was –15.1 ± 97.1 and 3.1 ± 2.7, respectively. The median FC value of decreased and increased transcripts was –2 and 2.1, respectively. Functional analysis revealed that genes related to monocyte differentiation are overrepresented among the DEGs (Table 2). Among the genes showing decreased expression in RetSat-null macrophages, we found only one related to phagocytosis of apoptotic cells; this was MFG-E8 [5], the expression of which was about 2.5 times less than that of the wild types cells (Table 1). To verify the finding, we determined the mRNA expression of MFG-E8 in BMDMs, peritoneal, and thioglycolate-elicited RetSat-null macrophages by qRT-PCR and found decreased expression (Figure 3A) as compared to wild type ones. Testing in BMDMs, we found that exposure to apoptotic cells for 5 h did not increase the expression of MFG-E8, but the initial difference in MFG-E8 expression remained constant (Figure 3B).

Since our initial analysis indicated that the expression of some differentiation-related genes was altered in RetSat-null macrophages (Table 2), we decided to determine whether MFG-E8 expression is altered during the differentiation of monocytes and whether administration of dihydroretinol alters it. As shown in Figure 3C, there was no difference in the expression of MFG-E8 of wild type and RetSat-null monocytes, and the expression of both RetSat and MFG-E8 was induced during monocyte differentiation. However, the induction of MFG-E8 expression was less pronounced in RetSat-null cells. Surprisingly, administration of dihydroretinol could not overcome the defect in the induction of MFG-E8. These data indicate that functions of RetSat other than dihydroretinol production during monocyte/macrophage differentiation regulates the expression of MFG-E8 in macrophages.

Though we did not find a difference in the expression of the retinoid-related genes in the RetSat-null BMDMs (Table 1), we also determined whether the administration of dihydroretinol during their differentiation affected their expression. As shown in Figure 3C, the expression of TG2 was induced during monocyte differentiation, and administration of dihydroretinol significantly induced its expression. However, we did not find any difference in the TG2 expression or in the dihydroretinol response of wild type and RetSat-null cells. The expression of the other four retinoid-regulated genes, on the other hand, was not induced by dihydroretinol during differentiation either in wild type or in RetSat-null cells (Figure 3D). Interestingly, despite of the lack of induction of these genes, dihydroretinol added during differentiation enhanced the short-term phagocytosis of macrophages, demonstrated by both FACS analysis and confocal microscopy (Figure 3E). However, since we did not find a difference in the short-term phagocytosis of wild type and RetSat-null BMDMs without adding dihydroretinol, these data indicate that despite the fact that RetSat expression is induced during monocyte/macrophage differentiation, dihydroretinoids might not have been produced in significant amounts.

Since we previously detected the induction of efferocytosis-related genes by retinoic acids in differentiated macrophages [21], we also tested their response to 24 h dihydroretinol treatment. Again, we detected a significant increase only in the expression of TG2 (2.8-fold), but not in that of the other retinoid-sensitive genes in both types of macrophages (data not shown).

Since we found that neuropeptide Y (NPY) is hardly expressed by RetSat-null macrophages (Table 1) but NPY was reported to promote the M2 phenotypic change of macrophages [35], which is known to be associated with enhanced phagocytic capacity [36], we also tested whether lack of NPY expression and MFG-E8 production could be related. We found (Figure 3C) that NPY expression is also induced during the differentiation of wild type monocytes, but surprisingly, it was downregulated during the differentiation of RetSat-null monocytes. Administration of dihydroretinol did not alter this pattern. We also tested whether administration of NPY could restore the defect in MFG-E8 expression during monocyte differentiation. However, administration of the neuropeptide at three different concentrations (10^−10^, 10^−9^, 10^−8^ M) to trigger different NPY receptors [37] did not alter the MFG-E8 expression either in wild type or in RetSat-null cells (data not shown). Interestingly, we could not detect a defect in NPY expression in other tissues tested.

### 3.4. Lower MFG-E8 Production Is Responsible for the Defect in Long-Term Phagocytosis of RetSat-Null Macrophages

**Figure 4:**
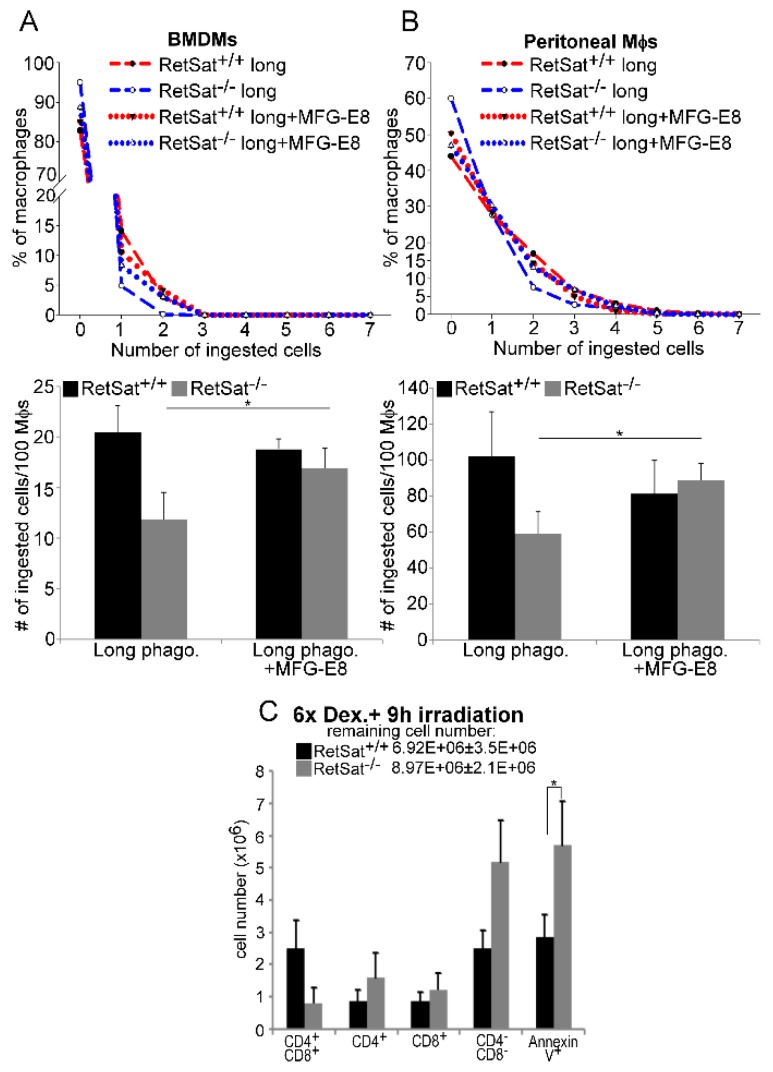
Impaired efferocytosis of RetSat-null macrophages is related to improper MFG-E8 production. Long-term efferocytosis of wild type and RetSat-null (**A**) BMDMs and (**B**) peritoneal macrophages was determined in the presence and absence of 2 μg/mL recombinant MFG-E8 protein. CFDA-SE-labelled macrophages were exposed to Deep Red dye-labelled apoptotic thymocytes for 1 h after continuous engulfing of non-labelled apoptotic thymocytes for 5 h. The number of ingested cells was determined by confocal microscopy. Upper panels show one representative experiment; total ingested cell number results in lower panels are expressed as mean ± SD (n = 3). (**C**) 4 week old mice were injected daily i.p. daily with 1 mg/kg DEX for 6 days to induce MFG-E8 expression and then exposed to 0.5 Gy irradiation to trigger thymocyte apoptosis. After 8 h, thymi were collected and the number of various thymocyte cell populations was determined by FACS analysis. Results are expressed as mean ± SD (n = 4), * *p* < 0.05.

To prove that decreased MFG-E8 production is responsible for the defect in long-term efferocytosis of RetSat-null macrophages, their long-term efferocytosis was tested in the absence and presence of recombinant MFG-E8. As shown in Figure 4A,B, administration of recombinant MFG-E8 restored the defect in long-term efferocytosis of both RetSat-null BMDMs and peritoneal macrophages, while it had no effect on the efferocytosis of wild type cells. These data indicate that the impaired long-term phagocytosis of apoptotic cells detected in vitro is indeed related to an impaired MFG-E8 production by RetSat-null macrophages.

Previous studies have shown that MFG-E8 is not expressed by thymic macrophages, thus it is very likely not involved in the clearance of apoptotic thymocytes [38], probably explaining why we did not see an efferocytosis-related phenotype in the thymus of RetSat-null mice. However, it was also reported that MFG-E8-null mice show an efferocytosis-related phenotype in their thymus if they are treated for a week with a daily low dose of DEX in order to induce MFG-E8 expression in macrophages and only then are exposed to X-ray irradiation to induce thymic apoptosis [29]. We repeated these experiments by using RetSat-null mice (Figure 4C) and found a significant increase both in the remaining total number and in the number of annexin-V-positive cells following irradiation, indicating accumulation of uncleared apoptotic thymocytes in the absence of RetSat.

### 3.5. Female RetSat-Null Mice Are Prone To Develop Mild Systemic Lupus Erythematosus (SLE)-Like Autoimmunity

It is well known that impaired clearance of apoptotic cells is associated with increased sensitivity to develop SLE-like autoimmunity [39]. This is related to the fact that uncleared apoptotic cells undergo secondary necrosis and induce inflammation. Their released autoantigens also induce autoantibody formation, demonstrated by repeatedly injecting apoptotic cells into mice [40]. In addition, proper phagocytosis should induce various anti-inflammatory mechanisms that become disturbed when efferocytosis is impaired. Efferocytosis receptor- or bridging-molecule-null mice are characterized by splenomegaly, anti-DNA and anti-nuclear autoantibodies, and immune complex deposits into the kidney, leading to glomerulonephritis [39]. Like humans, female mice are more prone to develop autoimmunity than male mice [41]. If loss of RetSat impairs the in vivo clearance of apoptotic cells by macrophages, it is expected that RetSat-null female mice are also more prone to develop SLE-like autoimmunity. Thus, we decided to investigate 1 year old female mice and compared them to age-matched wild type controls.

**Figure 5:**
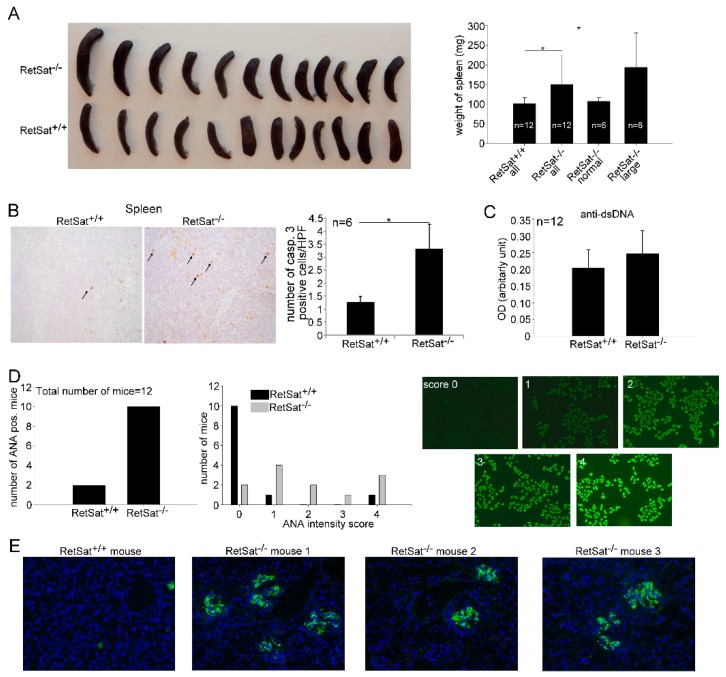
Aged female RetSat-null mice develop mild autoimmunity. (**A**) Splenic weights of 1 year old female wild type and RetSat-null mice. * *p* < 0.05. (**B**) Immunohistochemical detection of activated caspase-3-positive cells in the spleens of 6 wild type and the 6 RetSat-null mice with enlarged spleens was carried out as described in Materials and Methods. Data represent mean ± SD of the number of activated caspase-3-positive cells/high power field, * *p* < 0.05. (**C**) Levels of anti-dsDNA antibodies in the serum of wild type and RetSat-null mice are expressed as mean ± SD. OD; optical density. (**D**) Anti-nuclear antibodies (ANA) in the serum of 12 wild type and 12 RetSat-null mice. Images demonstrate the fluorescence intensity for the ANA scores from 0 to 4. (**E**) Immune complex deposits (shown with green color) in the kidneys of aged female RetSat-null mice. Cell nuclei are stained blue with 4′,6-diamidino-2-phenylindole (DAPI).

As seen in Figure 5A, the average spleen weight of RetSat-null female mice was significantly higher than that of the wild type females, and out of the 12 RetSat-null mice, 6 had bigger spleen weights than the average of the controls. Previous studies have shown that enlarged spleens in MFG-E8 null mice are associated with impaired clearance of apoptotic cells by tingible body macrophages that engulf apoptotic B cells generated in the germinal centers [38]. If apoptotic cells are not cleared properly, they remain for a longer time in the tissue and the number of activated caspase-3-positive cells increases. Thus, we determined the occurrence of activated caspase-3-positive cells in the spleens and found that it is significantly increased in the large spleens as compared to their wild type controls (Figure 5B). While we could not detect significantly increased anti-dsDNA antibody titers (Figure 5C), 10 out of the 12 RetSat-null mice produced anti-nuclear antibodies (Figure 5D). Immune complex deposits could easily be detected in the kidneys of mice with enlarged spleens, while no immune complex deposits could be detected in six randomly selected wild type kidneys (Figure 5E). Glomerular dysfunction was indicated in the mice with enlarged spleens by high serum concentrations of urea (52 to > 100 mM versus the normal 11 ± 3 mM).

## 4. Discussion

Increasing evidence indicates that in mammals, improper clearance of apoptotic cells sensitizes for the development of chronic inflammatory diseases and autoimmunity [39]. Thus, understanding the mechanisms that control proper efferocytosis in order to be able to modify them might contribute to our future handling of chronic inflammatory diseases [42]. Previous studies from our laboratory have shown that during engulfment of apoptotic cells, retinoids are formed in a retinaldehyde dehydrogenase-dependent manner that upregulate various efferocytosis-related genes, leading to enhanced phagocytic capacity during long-term efferocytosis. Our data also indicated that the retinoids formed might be products of the RetSat pathway. Indeed, we were able to demonstrate the induction of RetSat enzyme both in vivo in the mouse thymus following thymocyte apoptosis induction and in macrophages following LXR activation [21], though the expression of the enzyme in macrophages is about 60x and 20x less than in the liver and kidney, the two tissues that express the highest amount of RetSat, respectively [43]. In the present study, it was investigated whether loss of RetSat in mice affects efferocytosis in vitro and in vivo. The data presented in this paper indicate that macrophages differentiating in the absence of RetSat produce significantly less MFG-E8 than their wild type counterparts.

MFG-E8 is a 66 kDa glycoprotein that contains a signal sequence for secretion, two N-terminal epidermal growth factor (EGF) domains, and two C-terminal discoidin domains with homology to the C1 and C2 domains found in blood-clotting factors V and VIII. The second EGF domain contains an arginine–glycine–aspartic (RGD) integrin-binding motif that engages α_v_β_3_/α_v_β_5_ integrins to facilitate integrin-mediated signal transduction, while the C-terminal discoidin domains mediate attachment to PS on apoptotic cells [44]. Thus MFG-E8 acts as a bridging molecule for the α_v_β_3_/α_v_β_5_ integrins during efferocytosis, and its integrin β_3_ binding is further promoted by TG2, which acts as a coreceptor for integrin β_3_ [14]. Loss of MFG-E8 leads to development of autoimmunity that is specifically due to defects in apoptotic cell engulfment by tangible body macrophages in germinal centers [6,38].

The data presented by us demonstrate that macrophages from RetSat-null mice behave similarly to that of MFG-E8 null macrophages. In short-term efferocytosis experiments, they do not show efferocytosis defects, as was reported for the MFG-E8 null mice [45], but they have impaired efferocytosis in the long term. We believe that this might be related to the fact that MFG-E8 secretion is accelerated upon apoptotic cell exposure. In accordance, administration of recombinant MFG-E8 did not affect long-term efferocytosis by wild type macrophages, but it enhanced efferocytosis by RetSat-null BMDMs and peritoneal macrophages. In addition, as found with MFG-E8 null mice, a delayed efferocytosis was detected in the thymi of RetSat-null mice following X-ray exposure if they were pre-exposed to a daily dose of DEX for a week to induce MFG-E8 expression [29].

Like MFG-E8-null mice [6,38], aged female RetSat-null mice also develop SLE-like autoimmunity characterized by enlarged spleens, accumulation of apoptotic cells in the spleen, and anti-nuclear antibodies and immune complex deposits in the kidneys. Interestingly, aged TG2 (the integrin β_3_ coreceptor that promotes MFG-E8 binding)-null mice have a similar phenotype [46]. In the absence of proper clearance, autoimmunity develops not only due to the accumulation of apoptotic cells that undergo secondary necrosis, but also because uptake of apoptotic cells induces various anti-inflammatory mechanisms that also become impaired. In this respect, it is worth noting that both MFG-E8 [38] and NPY [47], which is not produced by RetSat-null macrophages, have anti-inflammatory properties. In addition, engulfing macrophage-derived MFG-E8 strongly contributes to the M2 polarization of the inflammatory macrophages [48], and proper macrophage M2 polarization is a requirement for the timed inflammatory resolution process [49,50]. Interestingly, RetSat-null macrophages also express lower endogenous CSF-1 and Gpr68/OGR1 (Table 1), and this might also decrease their M2 polarization potential as CSF-1 promotes macrophage differentiation into the M2 direction [51], while Gpr68-deficient macrophages express fewer M2 markers [52].

Increasing evidence indicates that MFG-E8 bound to α_v_β_3,5_ integrins not only promotes efferocytosis but is strongly coupled to fat metabolism as well. Thus, MFG-E8 enhances fatty acid uptake [53] and is also involved in the regulation of enterocyte lipid metabolism [54]. In the lipid turnover of the adipose tissue, adipose tissue macrophages continuously take up and metabolize lipids released from adipocytes in the form of exosomes [55]. Exosomes, by being PS positive, also utilize MFG-E8 to promote their cellular uptake [56]. In this context, it is worth noting that RetSat-null mice show increased adiposity [28]. It is interesting to speculate whether this phenotype could also be related to a decreased MFG-E8 production by adipose tissue macrophages by lowering the amount of exosomal lipids taken up and metabolized by them.

It has been shown that the expression of MFG-E8 is induced by granulocyte/monocyte colony-stimulating factor in macrophages [50]. We also detected the induction of MFG-E8 mRNA expression during monocyte differentiation, but the induction in RetSat-null cells was less than in wild type macrophages. We tested the role of dihydroretinol and found that the reduced expression of the gene is not related to dihydroretinol production by RetSat during this late differentiation process. Our data indicate that RetSat itself or its activity might contribute to the general expression level of MFG-E8, without affecting its inducibility. Interestingly, in RetSat-null macrophages we found a significantly decreased expression of GATA2 (4.5-fold), a zinc finger transcription factor that contributes to the proper monocyte/macrophage differentiation process [57], as well as that of C/EBPβ (Table 1). GATA2 was shown to regulate C/EBPβ expression [58], while C/EBPβ enhances MFG-E8 transcription [59].

In addition, we found that not only MFG-E8 but other differentiation-related genes are affected by the loss of RetSat (Table 1). Since we did not see evidence for dihydroretinol production during monocyte/macrophage differentiation, we believe that RetSat or its products might affect macrophage differentiation at an earlier phase, and thus, they might influence the differentiation of other monocyte-derived cells as well. In this context, it is worth noting that the expression of the dendritic cell-specific transmembrane protein (DC-STAMP) is also lower in RetSat-null macrophages (Table 1). We did not determine the expression of DC-STAMP in monocyte-derived dendritic cells, but DC-STAMP-null mice are also more sensitive to the development of autoimmunity, very likely due to altered dendritic cell functions [60]. Thus, we cannot exclude that a possibly altered DC-STAMP expression in dendritic cells might also contribute to the development of autoimmunity in RetSat-null mice.

## 5. Conclusions

Cumulatively, our data indicate that RetSat affects the differentiation of macrophages, but it might not be related to its ability to produce dihydroretinol, at least not during the monocyte/macrophage differentiation phase. Though administration of dihydroretinol during differentiation could enhance the short-term phagocytic capacity of macrophages, this was not related to the induction of retinoic acid receptor-regulated phagocytic genes, despite the fact that dihydroretinoids can selectively activate RAR and RXR receptors [23]. Since RetSat was induced during our monocyte differentiation protocol, if dihydroretinol was produced, it should have enhanced the short-term efferocytotic capacity of wild type macrophages. Since, however, loss of RetSat did not affect the short-term phagocytic capacity of macrophages generated and we did not find a difference in the expression of TG2, a dihydroretinol-sensitive gene, our observations indicate the lack of significant dihydroretinol production during monocyte differentiation at least during the in vitro protocol. However, we described an efferocytosis-related phenotype of these mice, which seems to be related to less efficient MFG-E8 and perhaps also to the lack of neuropeptide Y production by RetSat-null macrophages.

## Figures and Tables

**Table 1:** List of 117 differentially expressed genes (DEGs) between RetSat^+/+^ and RetSat^−/−^ BMDMs (based on at least a 1.5-fold change (FC) and corrected *p* (*p* (Corr)) value < 0.05).

Gene Symbol	FC	*p* (Corr)	Gene Symbol	FC	*p* (Corr)
Npy	−742.1	1.6 × 10^−05^	Dgat2	19.0	1.3 × 10^−04^
1700112E06Rik	−9.2	1.4 × 10^−04^	Nbea	8.2	6.9 × 10^−04^
Rtn4rl2	−5.4	2.6 × 10^−02^	Morn4	7.8	3.8 × 10^−06^
Dcstamp	−4.7	1.4 × 10^−02^	2410066E13Rik	7.4	1.3 × 10^−06^
Itgb7	−4.7	2.0 × 10^−02^	Fah	6.7	7.2 × 10^−08^
Gata2	−4.5	1.5 × 10^−02^	Hddc3	5.9	7.1 × 10^−07^
Wdr54	−3.8	1.4× 10^−02^	Wdfy1	5.8	2.2 × 10^−07^
Elmod3	−3.2	3.2 × 10^−04^	Dkk2	4.9	2.7 × 10^−05^
Gm15446	−2.9	5.3 × 10^−03^	Cdo1	4.4	1.9 × 10^−04^
Gm7799	−2.9	5.0 × 10^−02^	Sh3d21	4.4	6.8 × 10^−06^
Gpr68	−2.9	4.8 × 10^−02^	Slco3a1	4.3	8.4 × 10^−09^
Krt80	−2.9	2.5 × 10^−02^	AC166773.1	3.9	4.1 × 10^−06^
Mfge8	−2.7	2.4 × 10^−02^	Gbp10	3.6	1.7 × 10^−03^
Pde1c	−2.6	7.1 × 10^−03^	Gpx3	3.5	2.6 × 10^−04^
Galnt9	−2.5	3.5 × 10^−02^	Gm13014	3.5	3.0 × 10^−04^
Gm12895	−2.5	2.9 × 10^−02^	Camk2b	3.5	1.6 × 10^−03^
Gm14109	−2.5	2.6 × 10^−03^	Pde2a	3.2	4.4 × 10^−07^
Gm9746	−2.4	2.5 × 10^−04^	Lrrc9	3.2	7.3 × 10^−04^
Kalrn	−2.4	4.0 × 10^−02^	Itm2a	3.0	2.9 × 10^−04^
Il1rn	−2.4	1.8 × 10^−02^	Xkr6	2.9	7.7 × 10^−04^
Itpka	−2.3	5.4 × 10^−05^	Rab4a	2.7	5.3 × 10^−07^
Dnajb13	−2.3	3.5 × 10^−02^	Pyroxd2	2.7	8.0 × 10^−04^
Tnip3	−2.2	1.0 × 10^−02^	AC123679.1	2.6	8.2 × 10^−06^
Gpc1	−2.2	2.0 × 10^−02^	Efcab7	2.4	3.6 × 10^−04^
RP24-281K23.1	−2.1	7.1 × 10^−03^	Prdm5	2.3	1.7 × 10^−04^
Gm3788	−2.1	1.0 × 10^−02^	Mageh1	2.2	9.8 × 10^−04^
Gm9844	−2.1	1.0 × 10^−02^	Mlph	2.2	1.5 × 10^−03^
Tmsb10	−2.1	1.9 × 10^−02^	Gm4772	2.2	3.8 × 10^−04^
4931413K12Rik	−2.0	1.4 × 10^−02^	Nnt	2.1	7.3 × 10^−07^
D14Ertd449e	−2.0	1.8 × 10^−03^	Dhcr24	2.1	5.0 × 10^−05^
RetSat	−2.0	2.9 × 10^−04^	Syp	2.1	1.6 × 10^−03^
RP23-291E6.5	−2.0	2.9 × 10^−02^	Klra3	2.1	1.4 × 10^−05^
Itgax	−1.9	4.0 × 10^−02^	B130055M24Rik	2.0	6.3 × 10^−04^
Ak4	−1.9	3.0 × 10^−03^	C1qtnf6	2.0	6.1 × 10^−05^
Csf1	−1.9	1.4 × 10^−02^	Pdgfc	2.0	7.6 × 10^−05^
Tiam1	−1.9	3.3 × 10^−02^	AL627077.2	2.0	1.0 × 10^−03^
Maff	−1.9	1.0 × 10^−02^	Gm13228	2.0	9.8 × 10^−09^
Cd52	−1.9	2.9 × 10^−03^	Spr-ps1	2.0	5.8 × 10^−04^
Zfp932	−1.8	1.6 × 10^−02^	Neo1	1.9	9.7 × 10^−04^
Gm11625	−1.8	3.4 × 10^−02^	Tmem231	1.9	4.7 × 10^−04^
Ide	−1.7	7.2 × 10^−04^	Fam115a	1.9	6.3 × 10^−04^
Dfna5	−1.7	1.4 × 10^−02^	Usp11	1.8	9.5 × 10^−04^
Sema4a	−1.7	1.2 × 10^−02^	Aldh1l1	1.8	4.6 × 10^−04^
Cldn26	−1.7	1.7 × 10^−02^	Zcchc14	1.8	1.2 × 10^−03^
Lancl2	−1.7	7.1 × 10^−04^	Dynlt1b	1.8	6.8 × 10^−04^
Blm	−1.7	4.3 × 10^−02^	Dynlt1a	1.8	8.3 × 10^−04^
Oit3	−1.7	4.0 × 10^−02^	Ric3	1.7	6.4 × 10^−04^
Cebpb	−1.7	3.1 × 10^−02^	Slc40a1	1.7	7.9 × 10^−05^
Hilpda	−1.6	1.3 × 10^−02^	Ppp1r9a	1.7	2.6 × 10^−04^
S100a8	−1.6	2.5 × 10^−03^	Pbxip1	1.7	8.5 × 10^−04^
Padi4	−1.6	1.9 × 10^−02^	Dynlt1-ps1	1.6	1.5 × 10^−04^
Ephx1	−1.6	1.3 × 10^−02^	Cd59a	1.6	1.2 × 10^−05^
Gm7665	−1.5	4.5 × 10^−02^	Ms4a6b	1.6	4.9 × 10^−05^
Entpd4	−1.5	2.9 × 10^−04^	Sass6	1.6	1.3 × 10^−04^
Gm11810	−1.5	4.2 × 10^−02^	Plekha8	1.6	3.9 × 10^−04^
Napsa	−1.5	3.1 × 10^−02^	Cadm1	1.6	1.4 × 10^−03^
Ifi30	−1.5	1.1 × 10^−02^	Nradd	1.6	4.4 × 10^−04^
Gm12854	−1.5	1.5 × 10^−02^	AC113059.1	1.5	1.1 × 10^−03^
Gm10108	−1.5	1.9 × 10^−02^			

Data were generated from BMDMs differentiated from 3–3 wild type or RetSat-null mice. Please note that we detect some RetSat mRNA expression in RetSat-null cells as well. This is related to the fact that during the generation of the knock-out mice, only exon 1 of the RetSat gene was replaced by a neomycin resistance cassette, and it is transcribed together with the remaining exons, producing a non-functional RetSat mRNA.

**Table 2:** Gene ontology (GO) biological process overrepresentation test of the 117 DEGs. FDR: false discovery rate value calculated by the Benjamini–Hochberg procedure for multiple test correction (FDR < 0.05).

GO-Term	Description	Count in Gene Set	FDR
GO:0061003	positive regulation of dendritic spine morphogenesis	4 of 23	0.0107
GO:0036006	cellular response to macrophage colony-stimulating factor stimulus	3 of 8	0.0126
GO:0045655	regulation of monocyte differentiation	3 of 17	0.0294
GO:0032502	developmental process	40 of 5213	0.0294

## References

[1] Arandjelovic S., Ravichandran K.S. (2015). Phagocytosis of apoptotic cells in homeostasis. Nat. Immunol..

[2] Fadok V.A., Henson P.M. (2003). Apoptosis: Giving phosphatidylserine recognition an assist--with a twist. Curr. Biol..

[3] Park D., Tosello-Trampont A.C., Elliott M.R., Lu M., Haney L.B., Ma Z., Klibanov A.L., Mandell J.W., Ravichandran K.S. (2007). BAI1 is an engulfment receptor for apoptotic cells upstream of the ELMO/Dock180/Rac module. Nature.

[4] Park S.Y., Jung M.Y., Kim H.J., Lee S.J., Kim S.Y., Lee B.H., Kwon T.H., Park R.W., Kim I.S. (2008). Rapid cell corpse clearance by stabilin-2, a membrane phosphatidylserine receptor. Cell Death Differ..

[5] Miyanishi M., Tada K., Koike M., Uchiyama Y., Kitamura T., Nagata S. (2007). Identification of Tim4 as a phosphatidylserine receptor. Nature.

[6] Hanayama R., Tanaka M., Miwa K., Shinohara A., Iwamatsu A., Nagata S. (2002). Identification of a factor that links apoptotic cells to phagocytes. Nature.

[7] Savill J.S., Hogg N., Ren Y., Haslett C. (1992). Thrombospondin cooperates with CD36 and the vitronectin receptor in macrophage recognition of neutrophils undergoing apoptosis. J. Clin. Invest..

[8] Stitt T.N., Conn G., Gore M., Lai C., Bruno J., Radziejewski C., Mattsson K., Fisher J., Gies D.R., Jones P.F. (1995). The anticoagulation factor protein S and its relative, Gas6, are ligands for the Tyro 3/Axl family of receptor tyrosine kinases. Cell.

[9] Botto M., Dell’Agnola C., Bygrave A.E., Thompson E.M., Cook H.T., Petry F., Loos M., Pandolfi P.P., Walport M.J. (1998). Homozygous C1q deficiency causes glomerulonephritis associated with multiple apoptotic bodies. Nat. Genet..

[10] Park D., Hochreiter-Hufford A., Ravichandran K.S. (2009). The phosphatidylserine receptor TIM-4 does not mediate direct signaling. Curr. Biol..

[11] Devitt A., Parker K.G., Ogden C.A., Oldreive C., Clay M.F., Melville L.A., Bellamy C.O., Lacy-Hulbert A., Gangloff S.C., Goyert S.M. (2004). Persistence of apoptotic cells without autoimmune disease or inflammation in CD14−/− mice. J. Cell. Biol..

[12] Greenberg M.E., Sun M., Zhang R., Febbraio M., Silverstein R., Hazen S.L. (2006). Oxidized phosphatidylserine-CD36 interactions play an essential role in macrophage-dependent phagocytosis of apoptotic cells. J. Exp. Med..

[13] Cohen P.L., Caricchio R., Abraham V., Camenisch T.D., Jennette J.C., Roubey R.A., Earp H.S., Matsushima G., Reap E.A. (2002). Delayed apoptotic cell clearance and lupus-like autoimmunity in mice lacking the c-Mertk membrane tyrosine kinase. J. Exp. Med..

[14] Tóth B., Garabuczi E., Sarang Z., Vereb G., Vámosi G., Aeschlimann D., Blaskó B., Bécsi B., Erdődi F., Lacy-Hulbert A. (2009). Transglutaminase 2 is needed for the formation of an efficient phagocyte portal in macrophages engulfing apoptotic cells. J. Immunol..

[15] Kinchen J.M., Cabello J., Klingle D., Wong K., Freichtinger R., Schnabel H., Schnabel R., Hengartner M.O. (2005). Two pathways converge at CED-10 to mediate actin rearrangement and corpse removal in C. elegans, Nature.

[16] Miksa M., Amin D., Wu R., Jacob A., Zhou M., Dong W., Yang W.L., Ravikumar T.S., Wang P. (2008). Maturation-induced down-regulation of MFG-E8 impairs apoptotic cell clearance and enhances endotoxin response. Int. J. Mol. Med..

[17] Noelia A., Bensinger S.J., Hong C., Beceiro S., Bradley M.N., Zelcer N., Deniz J., Ramirez C., Díaz M., Gallardo G. (2009). Apoptotic cells promote their own clearance and immune tolerance through activation of the nuclear receptor LXR. Immunity.

[18] Roszer T., Menéndez-Gutiérrez M.P., Lefterova M.I., Alameda D., Núñez V., Lazar M.A., Fischer T., Ricote M. (2011). Autoimmune kidney disease and impaired engulfment of apoptotic cells in mice with macrophage peroxisome proliferator-activated receptor gamma or retinoid X receptor alpha deficiency. J. Immunol..

[19] Mukundan L., Odegaard J.I., Morel C.R., Heredia J.E., Mwangi J.W., Ricardo-Gonzalez R.R., Goh Y.P., Eagle A.R., Dunn S.E., Awakuni J.U. (2009). PPAR-delta senses and orchestrates clearance of apoptotic cells to promote tolerance. Nat. Med..

[20] Garabuczi É., Kiss B., Felszeghy S., Tsay G.J., Fésüs L., Szondy Z. (2013). Retinoids produced by macrophages engulfing apoptotic cells contribute to the appearance of transglutaminase 2 in apoptotic thymocytes. Amino Acids.

[21] Sarang Z., Joós G., Garabuczi É., Rühl R., Gregory C.D., Szondy Z. (2014). Macrophages engulfing apoptotic cells produce nonclassical retinoids to enhance their phagocytic capacity. J. Immunol..

[22] Moise A.R., Domínguez M., Alvarez S., Alvarez R., Schupp M., Cristancho A.G., Kiser P.D., de Lera A.R., Lazar M.A., Palczewski K. (2008). Stereospecificity of retinol saturase: Absolute configuration, synthesis, and biological evaluation of dihydroretinoids. J. Am. Chem. Soc..

[23] Moise A.R., Alvarez S., Domínguez M., Alvarez R., Golczak M., Lobo G.P., von Lintig J., de Lera A.R., Palczewski K. (2009). Activation of retinoic acid receptors by dihydroretinoids. Mol. Pharmacol..

[24] Krężel W., Rühl R., de Lera A.R. (2019). Alternative retinoid X receptor (RXR) ligands. Mol. Cell. Endocrinol..

[25] Schupp M., Lefterova M., Janke J., Leitner K., Cristancho A.G., Mullican S.E., Qatanani M., Szwergold N., Steger D.J., Curtin J.C. (2009). Retinol saturase promotes adipogenesis and is downregulated in obesity. Proc. Natl Acad. Sci. USA..

[26] Pang X.Y., Wang S., Jurczak M.J., Shulman G.I., Moise A.R. (2017). Retinolsaturase modulates lipid metabolism and the production of reactive oxygen species. Arch. Biochem. Biophys..

[27] Heidenreich S., Witte N., Weber P., Goehring I., Tolkachov A., von Loeffelholz C., Döcke S., Bauer M., Stockmann M., Pfeiffer A.F.H. (2017). Retinolsaturase coordinates liver metabolism by regulating ChREBP activity. Nat. Commun..

[28] Moise A.R., Lobo G.P., Erokwu B., Wilson D.L., Peck D., Alvarez S., Domínguez M., Alvarez R., Flask C.A., de Lera A.R. (2010). Increased adiposity in the retinol saturase-knockout mouse. FASEB J..

[29] Lauber K., Keppeler H., Munoz L.E., Koppe U., Schröder K., Yamaguchi H., Krönke G., Uderhardt S., Wesselborg S., Belka C. (2013). Milk fat globule-EGF factor 8 mediates the enhancement of apoptotic cell clearance by glucocorticoids. Cell Death Differ..

[30] Rühl R. (2006). Method to determine 4-oxo-retinoic acids, retinoic acids and retinol in serum and cell extracts by liquid chromatography/diode-array detection atmospheric pressure chemical ionisation tandem mass spectrometry. Rapid. Commun. Mass Spectrum.

[31] Rahmatullah M., Boyde T.R. (1980). Improvements in the determination of urea using diacetyl monoxime; methods with and without deproteinisation. Clin. Chim. Acta..

[32] Starr T.K., Jameson S.C., Hogquist K.A. (2003). Positive and negative selection of T Cells. Ann. Rev. Immunol..

[33] Chen W., Konkel J.E. (2015). Development of thymic Foxp3(+) regulatory T cells: TGF-β matters. Eur. J. Immunol..

[34] Sarang Z., Garabuczi É., Joós G., Kiss B., Tóth K., Rühl R., Szondy Z. (2013). Macrophages engulfing apoptotic thymocytes produce retinoids to promote selection, differentiation, removal and replacement of double positive thymocytes. Immunobiology.

[35] Buttari B., Profumo E., Darcangelo D., Di Raimo T., Businaro R., Capoano R., Salvati B., Saso L., Elenkov I. (2017). Neuropeptide Y as regulator of macrophage phenotype and functions: A neuroimmune CUE in atherosclerosis regression?. Atherosclerosis.

[36] Zizzo G., Hilliard B.A., Monestier M., Cohen P.L. (2012). Efficient clearance of early apoptotic cells by human macrophages requires M2c polarization and MerTK induction. J. Immunol..

[37] Pedragosa-Badia X., Stichel J., Beck-Sickinger A.G. (2013). Neuropeptide Y receptors: How to get subtype selectivity. Front. Endocrinol..

[38] Hanayama R., Tanaka M., Miyasak K., Aozasa K., Koike M., Uchiyama Y., Nagata S. (2004). Autoimmune disease and impaired uptake of apoptotic cells in MFG-E8-deficient mice. Science.

[39] Abdolmaleki F., Farahani N., Gheibi Hayat S.M., Pirro M., Bianconi V., Barreto G.E., Sahebkar A. (2018). The Role of Efferocytosis in Autoimmune Diseases. Front. Immunol..

[40] Mevorach D., Zhou J.L., Song X., Elkon K.B. (1998). Systemic exposure to irradiated apoptotic cells induces autoantibody production. J. Exp. Med..

[41] Weckerle C.E., Niewold T.B. (2011). The Unexplained Female Predominance of Systemic Lupus Erythematosus: Clues from Genetic and Cytokine Studies. Clin. Rev. Allergy Immunol..

[42] Szondy Z., Garabuczi E., Joós G., Tsay G.J., Sarang Z. (2014). Impaired clearance of apoptotic cells in chronic inflammatory diseases: Therapeutic implications. Front. Immunol..

[43] GeneAtlas MOE430, gcrma. http://ds.biogps.org/?dataset=GSE10246&gene=67442.

[44] Matsuda A., Jacob A., Wu R., Zhou M., Nicastro J.M., Coppa G.F., Wang P. (2011). Milk fat globule-EGF factor VIII in sepsis and ischemia-reperfusion injury. Mol. Med..

[45] Michalski M.N., Seydel A.L., Siismets E.M., Zweifler L.E., Koh A.J., Sinder B.P., Aguirre J.I., Atabai K., Roca H., McCauley L.K. (2018). Inflammatory bone loss associated with MFG-E8 deficiency is rescued by teriparatide. FASEB J..

[46] Szondy Z., Sarang Z., Molnar P., Nemeth T., Piacentini M., Mastroberardino P.G., Falasca L., Aeschlimann D., Kovacs J., Kiss I. (2003). Transglutaminase 2−/− mice reveal a phagocytosis-associated crosstalk between macrophages and apoptotic cells. Proc. Natl. Acad. Sci. USA.

[47] Dimitrijević M., Stanojević S., Mitić K., Kustrimović N., Vujić V., Miletić T., Kovacević-Jovanović V. (2008). The anti-inflammatory effect of neuropeptide Y (NPY) in rats is dependent on dipeptidyl peptidase 4 (DP4) activity and age. Peptides.

[48] Soki F.N., Koh A.J., Jones J.D., Kim Y.W., Dai J., Keller E.T., Pienta K.J., Atabai K., Roca H., McCauley L.K. (2014). Polarization of prostate cancer-associated macrophages is induced by milk fat globule-EGF factor 8 (MFG-E8)-mediated efferocytosis. J. Biol. Chem..

[49] Atri C., Guerfali F.Z., Laouini D. (2018). Role of Human Macrophage Polarization in Inflammation during Infectious Diseases. Int. J. Mol. Sci..

[50] Jinushi M., Nakazaki Y., Dougan M., Carrasco D.R., Mihm M., Dranoff G. (2007). MFG-E8-mediated uptake of apoptotic cells by APCs links the pro- and antiinflammatory activities of GM-CSF. J. Clin. Invest..

[51] Martinez F.O., Gordon S., Locati M., Mantovani A. (2006). Transcriptional profiling of the human monocyte-to-macrophage differentiation and polarization: New molecules and patterns of gene expression. J. Immunol..

[52] Yan L., Singh L.S., Zhang L., Xu Y. (2014). Role of OGR1 in myeloid-derived cells in prostate cancer. Oncogene.

[53] Khalifeh-Soltani A., McKleroy W., Sakuma S., Cheung Y.Y., Tharp K., Qiu Y., Turner S.M., Chawla A., Stahl A., Atabai K. (2014). Mfge8 promotes obesity by mediating the uptake of dietary fats and serum fatty acids. Nat. Med..

[54] Khalifeh-Soltani A., Gupta D., Ha A., Iqbal J., Hussain M., Podolsky M.J., Atabai K. (2016). Mfge8 regulates enterocyte lipid storage by promoting enterocyte triglyceride hydrolase activity. JCI Insight..

[55] Flaherty S.E., Grijalva A., Xu X., Ables E., Nomani A., Ferrante A.W. (2019). A lipase-independent pathway of lipid release and immune modulation by adipocytes. Science.

[56] Raposo G., Stoorvogel W. (2013). Extracellular vesicles: Exosomes, microvesicles, and friends. J. Cell. Biol..

[57] Rodrigues N.P., Boyd A.S., Fugazza C., May G.E., Guo Y., Tipping A.J., Scadden D.T., Vyas P., Enver T. (2008). GATA-2 regulates granulocyte-macrophage progenitor cell function. Blood.

[58] Rui W., Jin Z., Zhe G., Song H. (2013). The methylation of C/EBP β gene promoter and regulated by GATA-2 protein. Mol. Biol. Rep..

[59] Aziz M.M., Ishihara S., Rumi M.A., Mishima Y., Oshima N., Kadota C., Moriyama I., Li Y.Y., Rahman F.B., Otani A. (2008). Prolactin induces MFG-E8 production in macrophages via transcription factor C/EBPbeta-dependent pathway. Apoptosis..

[60] Sawatani Y., Miyamoto T., Nagai S., Maruya M., Imai J., Miyamoto K., Fujita N., Ninomiya K., Suzuki T., Iwasaki R. (2008). The role of DC-STAMP in maintenance of immune tolerance through regulation of dendritic cell function. Int. Immunol..

